# Simultaneous interfacility transfer of multiple non-critically ill COVID-19 patients using a single vehicle: the ambulance bus experience

**DOI:** 10.1186/s12245-022-00415-7

**Published:** 2022-03-05

**Authors:** Dennis G. Barten, Remco van Zijl, Frank W. J. Körver, Nathalie A. L. R. Peters

**Affiliations:** 1 Department of Emergency Medicine, VieCuri Medical Center, P.O. Box 1926, 5900 BX Venlo, The Netherlands; 2 AmbulanceZorg Limburg-Noord, Venlo, The Netherlands; 3 GGD Zuid-Limburg, Heerlen, The Netherlands

**Keywords:** COVID-19, Pandemic, Emergency medical services, Ambulance, Evacuation, Interfacility transfer

## Abstract

**Background:**

During the COVID-19 pandemic, hospital capacity in the Netherlands has been pushed to its limits. In order to prevent hospitals from collapse due to capacity issues, hospitalized COVID-19 patients were redistributed throughout the country. The numerous individual interfacility transfers further increased the pressure on emergency medical services (EMS), which simultaneously had to serve the community during the pandemic. In this report, we evaluate the interfacility transport of multiple non-critically ill COVID-19 patients using one single vehicle: a coach converted into an ambulance bus.

**Discussion:**

Between March 28, 2020, and July 17, 2021, the ambulance bus was dispatched 22 times. In total, 102 patients were transferred over a mean distance of 79.6 km. No technical or patient-related adverse events were reported. The primary benefits of the ambulance bus were its time and staff reducing potential, as well as the ability to provide relief to overwhelmed hospitals. Furthermore, it could be assembled from existing equipment in a relatively short time span. However, the efficiency of dispatches and matching between hospitals could be improved.

**Conclusion:**

The simultaneous interfacility transfer of multiple non-critically ill COVID-19 patients using an ambulance bus was feasible. No technical or patient-related adverse events were reported during 22 dispatches, involving a total of 102 patients. This mode of transport may also be useful in non-pandemic situations, such as hospital and nursing home evacuations.

## Background

The COVID-19 pandemic has resulted in surging numbers of patients requiring hospital care all over the world [1]. Similar to many other countries, hospital capacity in the Netherlands has been pushed to its limits. In order to prevent hospitals from being overwhelmed by COVID-19 admissions and to equally distribute pressures between hospitals, a national task force (Landelijk Coördinatiecentrum Patiënten Spreiding; LCPS) was established which coordinated interfacility transfers of hospitalized COVID-19 patients in the Netherlands [2]. Depending on the severity of disease, types of transportation included advanced life support (ALS) ambulances, mobile intensive care units (MICUs), and helicopter emergency medical services (HEMS) [3, 4]. This complex operation required full transparency within the healthcare system as well as close cooperation between hospitals [2].

In the Netherlands, the first case of COVID-19 was confirmed on February 27, 2020. Soon thereafter, hospitalization rates increased dramatically. However, there were considerable regional differences with regard to case numbers and hospital admission rates, necessitating the transfer of large numbers of COVID-19 patients throughout the country. This further exacerbated the pressure on emergency medical services (EMS), which simultaneously had to serve the community during the pandemic.

The transport of multiple patients at once could possibly enhance EMS capacity. In this report, we evaluate our approach to the interfacility transport of multiple non-critically ill COVID-19 patients using one single vehicle: a coach converted into an ambulance bus.

### Setting

The Netherlands, a western European country that is home to 17.5 million people, is provided with a modern healthcare system with effective primary care and a finely meshed network of 83 acute care hospitals. Ambulance care is organized into 25 regional ambulance services, of which two serve the Limburg province (1,117,201 inhabitants) in the southeast of the Netherlands (Table 1) [5]. Dutch EMS are staffed by ALS trained nurses who have completed a nine to 8-month EMS fellowship [6].

**Table 1:** Emergency medical services in the Limburg province, the Netherlands (2019)*

Regional ambulance service	AmbulanceZorg Limburg-Noord	GGDZL
Adherence area	Northern Limburg	Southern Limburg
Ambulance stations	12	5
Ambulance vehicles	24	25
**Ambulance dispatches**	**38,619**	**52,360**
Category 1	20,019	24,541
Category 2	12,173	15,363
Category 3	6427	12,456

*Derived from: Ambulancezorg Nederland [5]. Category 1: emergency care level (A1): life-threatening situations; Category 2: emergency care level (A2): not life-threatening, but urgent response required; Category 3: non-emergency patient transport services (B)

In the Netherlands, there have been 2.8 million confirmed cases of infection (of which 84.243 were hospitalized) and 20.125 confirmed COVID-19 deaths as of December 13, 2021 [7]. The Limburg province was one of the regions with the highest prevalence of COVID-19 in the Netherlands, with COVID-19 admission rates ranging from 369 to 742 per 100,000 inhabitants. The nationwide redistribution of hospitalized COVID-19 patients by a national task force resulted in a large demand for interfacility transfers, often over longer distances [2]. This further exacerbated the already high burden on EMS during the pandemic.

### Intervention

The transport of multiple patients at once could possibly relieve the pressure on EMS. Therefore, several alternatives for separate interfacility transfers were assessed before the intervention was conceived. Using a converted bus as an ambulance is not an entirely new concept. In 2016, three ambulance regions in the Netherlands jointly started using an ambulance bus intended for on-scene treatment of critically ill patients in case of mass casualty incidents or disasters. This vehicle (VanHool A330, VanHool, Lier, Belgium, 2003) was constructed from an existing bus and has an intensive care unit capacity of 6 spaces [8]. Similarly, researchers from France describe how a long-distance bus was redesigned and equipped to accommodate up to 6 intensive care patients with COVID-19. This so-called Collective Critical Care Ambulance was successfully tested in a short-distance transport of 4 critically ill patients. However, safety data on long-distance transfers by this vehicle are not yet available [9]. Similar transportation modes exist in other countries and are primarily used for medical evacuation [10]. In addition, buses have been developed that can be used for repatriation of patients, including facilities to secure stretchers and to carry wheelchairs. All of these initiatives are targeted on smaller patient numbers or on treatment capacity at the (disaster) scene. Nevertheless, they inspired our ambulance service to develop an ambulance bus with the ability to safely transfer 4–6 hospitalized, non-critically ill patients with COVID-19.

The simultaneous transfer of multiple patients may have several benefits. It reduces the number of required ambulance vehicles (including staff), so that most of the transfers can be planned during office hours. Moreover, compared to individual patient transfers, it may reduce the total handover time of already overwhelmed hospitals.

Some requirements were set. First, the quality of care in the ambulance bus had to equal that of regular ambulance care and be safe for both patients and personnel. Second, to preserve EMS capacity, the innovation had to support the appropriate use of available resources. Third, a maximum of two “pick up” and two “drop off” locations was determined to prevent a so-called commuter train effect. Finally, the ambulance bus also had to be useful in non-pandemic situations, such as hospital or nursing home evacuations.

### Construction

**Fig. 1:**
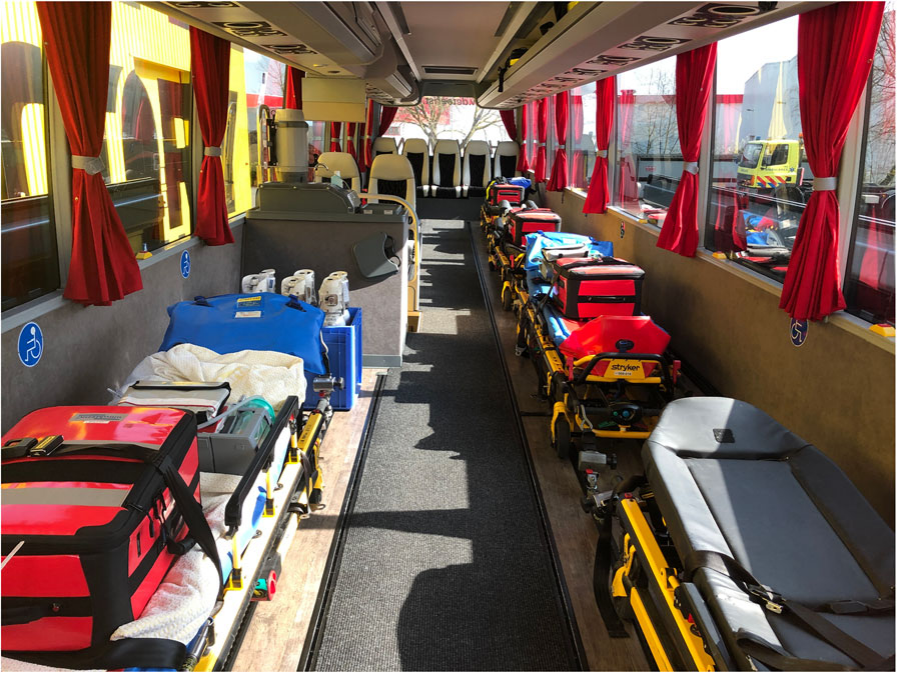
The interior of the ambulance bus

The bus was reconstructed from an existing coach (VanHool EX17 high, VanHool, Lier, Belgium, 2018). The rear of the vehicle was adjusted to fit a stretcher elevator (Dhollandia DH-RB.05, Dhollandia, Lokeren, Belgium), and the floor of the bus was fortified and provided with airline rails for the fixation of stretchers and wheelchairs. The Netherlands Vehicle Authority (Rijksdienst voor het Wegverkeer, RDW) approved the vehicle for wheelchair transport and supine position transfer of patients. An overview of medical equipment and personnel is provided in Table 2. The interior of the ambulance bus is shown in Fig. 1.

**Table 2:** Ambulance bus: medical equipment and personnel

**Technical supplies, per patient unit (*n*** **= 6)**	
Patient monitor and defibrillator (corpuls3, GS – Elektromedizinische Geräte, Kaufering, Germany)	
Advanced Life Support backpack^a^	
Oxygen backpack (oxygen cylinder, mayotubes, oxygen mask, bag-valve mask)	
Oxygen supply (1 x 2 L and 1 x 5 L)	
**Spare supplies**	
6 spare oxygen cylinders (6 x 2 L; 6 x 5 L)	
2 suction units	
2 respiratory monitors	
2 perfusion units	
**Personnel**	
2 bus drivers	
2 nurses (at least one ALS certified)	
**Personal protective materials**	
FFP2 face mask	
Goggles or face shield	
Disposable gown	
Gloves	

^a^Contains comprehensive EMS provision, including endotracheal intubation kit, supraglottic airway devices, cricothyroidotomy kit, infusion systems and fluids, intraosseous infusion materials, prehospital emergency medication set, and a myriad of needles, syringes, and bandages

### Staff

**Fig. 2:**
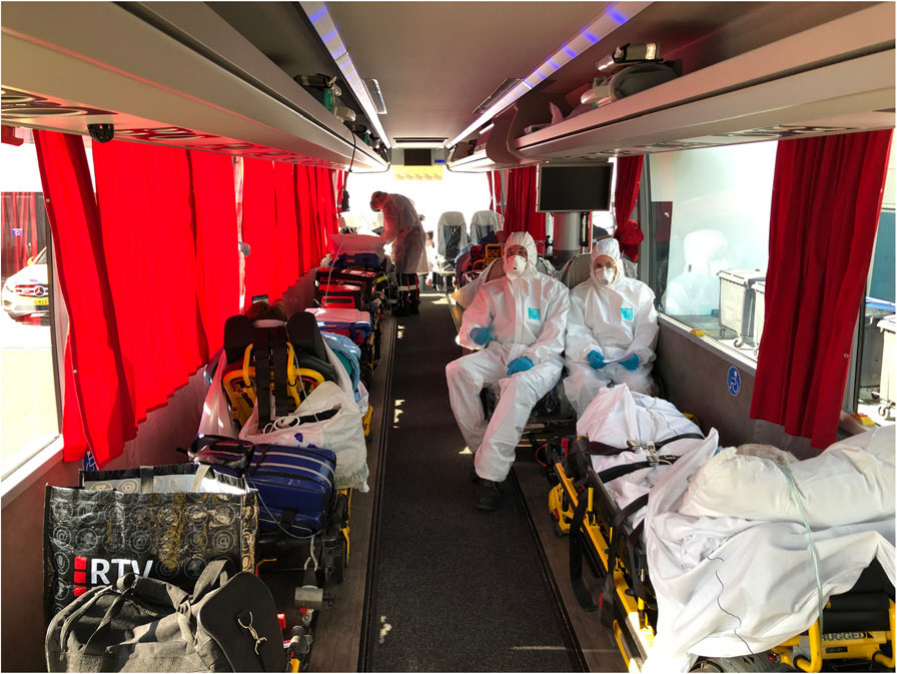
The interior of the ambulance with 2 ambulance nurses in position, wearing full PPM

**Fig. 3:**
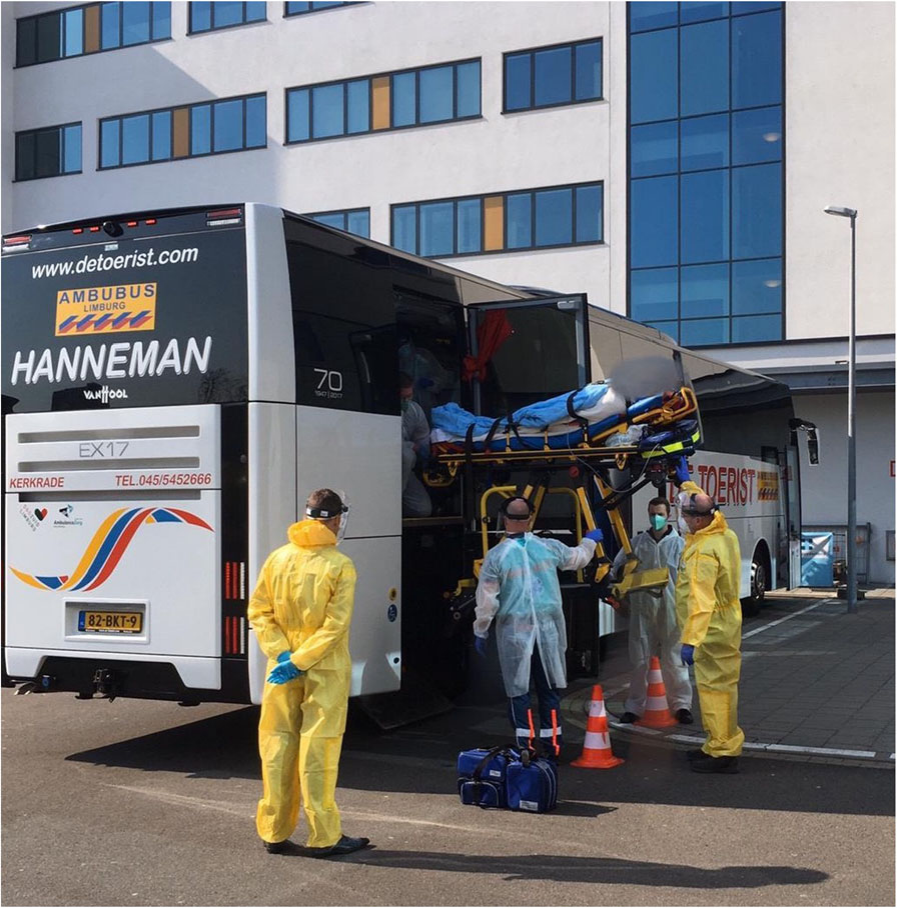
Unloading procedure of a patient

The ambulance bus was staffed with two bus drivers and two ambulance nurses. The two drivers were positioned on the driver’s and co-driver’s seat and drove the bus alternately. There was no barrier between the driver’s compartment and the cabin of the bus, but internal airflow was directed backwards with a high replenishment rate. Furthermore, the drivers wore full personal protective materials (PPM). The two ambulance nurses were positioned in the cabin (facing backwards) and wore full PPM as well (Fig. 2). Full PPM compromised a disposable gown, goggles, gloves, and FFP2 facemask. Before each dispatch, a team briefing was conducted. This briefing included tasks and responsibilities, how to deal with adverse events (technical failure of the bus or equipment; clinical deterioration) and the sequence of (un)loading of patients (Fig. 3).

### Hygienic procedure

In addition to the regular hygiene and disinfection procedure, the vehicle was cleaned with a 6% hydrogen peroxide solution using an aerial surface disinfection machine (Nocospray©, Oxy’Pharm, Champigny sur Marne, France). This procedure takes 40 min, including a mandatory ventilation period of 20 min.

### Indications

Patients were only eligible for ambulance bus transfers if they had a *proven* COVID-19 infection. Exclusion criteria were *suspected* COVID-19 infection, high-flow oxygen therapy, mechanical ventilation, and hemodynamic instability. Moreover, the expected oxygen demand should not exceed 50% of the total oxygen supply (which was set as safety margin). If the patient’s clinical condition would deteriorate during transport, and permanent individual ALS care would be required, the patient should be handed over to an ALS ambulance.

In case of clinical deterioration which necessitated permanent individual ALS care, such care could be initiated within the ambulance bus by the present nursing staff. Subsequently, there were 3 different scenarios to address such cases: (1) conveying the patient to the nearest hospital, (2) “rendezvous” with an ALS ambulance at the nearest parking area or highway exit, and (3) “rendezvous” with an ALS ambulance on the highway. At all times, police assistance could be requested by a portable transceiver.

### Coordination

Saturated hospitals could request to transfer eligible patients by announcing them to the national task force LCPS, which attempted to match patients with less crowded hospitals. In case of a match, the ambulance dispatch center was advised. Interfacility transfers by ambulance bus had to be endorsed by the crisis coordinator of the ambulance service. Patient handover information was exchanged between hospitals on beforehand, and ambulance personnel had essential patient information at one’s disposal too. For all transfers, informed consent from patients was required. The ambulance bus was not dispatched during night hours; startup time was approximately 5 h.

### Dispatches

Between March 28, 2020, and July 17, 2021, the ambulance bus was dispatched 22 times. In total, 102 patients were transferred and the mean distance of transfers was 79.6 km. No patient-related adverse events were reported.

In 2020 and 2021, the ambulance bus was also dispatched for non-pandemic emergencies. A major wildfire near the village Herkenbosch on April 21, 2020, necessitated the emergent evacuation of 92 residents of a nursing home [11]. Six of these residents were bedridden and evacuated by the ambulance bus. Furthermore, during the 2021 European floods, the River Maas reached its highest summertime level in over 100 years. This necessitated the evacuation of an entire hospital in the proximity of this river [12]. In total, 28 ambulance vehicles were deployed to transfer in-hospital patients to other facilities. The ambulance bus was dispatched to the hospital scene but remained stand-by only and was not used for the evacuation of patients.

### Evaluation

There was a team debriefing after each dispatch led by a crisis coordinator of the ambulance service, and after the first COVID-19 wave a thorough process evaluation took place. This yielded multiple lessons learned. First, the ambulance bus could be assembled from existing equipment in a relatively short time span. Hence, costs were limited and the ambulance bus could be swiftly deployed. Second, coordination between hospitals is essential to execute smooth operations. There only exists added value of the ambulance bus over conventional transport modes when 4 or more patients have to be transferred. To prevent redundant dispatches, the procedure should only be initiated if all transfer requests are endorsed and if informed consent is acquired from all patients. Third, the LCPS task force worked well for individual patient transfers, but was more challenging for the transfer of multiple patients. In general, the LCPS strived to equally alleviate the pressures on hospitals from day-to-day. This was at times conflicting with the optimal use of the ambulance bus, which is most efficient if multiple patients can be picked up at once in hospital A and have to be transferred to hospital B. Contrastingly, most patient transfers concerned the transfer of small patient numbers between multiple hospitals. Although no technical failures and patient-related adverse events were noted, the number of transfers and transferred patients were relatively low, limiting the ability to make final safety statements.

## Discussion

A largescale outbreak of an infectious disease, such as the ongoing COVID-19 pandemic, has the potential to overwhelm clinical hospital capacity in regions with high case numbers. It may therefore be necessary to distribute hospitalized patients to less crowed hospitals over longer distances. During the COVID-19 pandemic in the Netherlands, an ambulance bus was used to simultaneously transfer 4–6 non-critically ill COVID-19 patients to other hospitals. This innovative transport mode for COVID-19 patients was feasible. The primary benefits of the ambulance bus were its time and staff reducing potential, as well as the ability to provide relief to overwhelmed hospitals.

The first COVID-19 wave in the Netherlands (March–June 2020) especially took its toll in the southeast. As a result, hospital capacity shortages were unequally distributed throughout the country, whilst hospitals in the southeastern region were overwhelmed by COVID-19 admissions, relatively low numbers of patients were admitted to other hospitals in the country. As a result, there was room to redistribute hospitalized patients from the most saturated hospitals to less crowded hospitals in the country [13]. However, the second/third wave (July 2020–March 2021) was different. During this lengthy phase of the pandemic, case numbers were more evenly distributed throughout the country and all Dutch hospitals experienced similar high pressures. This phenomenon prohibited hospitals from receiving multiple patients at once, which reduced the usefulness of a vehicle such as the ambulance bus. Therefore, it seems to be most useful in situations where clinical capacity is unevenly saturated between regions.

The ambulance bus proved useful during the COVID-19 pandemic, but may also be deployed for other situations, such as hospital or nursing home evacuations. As shown by a previous report, a coach can also be converted into an ambulance bus which is equipped for the transport of multiple intensive care patients [9].

## Conclusions

The simultaneous interfacility transfer of multiple non-critically ill COVID-19 patients using an ambulance bus was feasible. No technical or patient-related adverse events were reported during 22 dispatches, involving a total of 102 patients. However, the efficiency of dispatches and matching between hospitals could be improved. This mode of transport may also be useful in non-pandemic situations, such as hospital and nursing home evacuations.

## Data Availability

Not applicable.

## Abbreviations

ALS: Advanced life support
EMS: Emergency medical services
HEMS: Helicopter emergency medical services
LCPS: Landelijk Coördinatiecentrum Patiënten Spreiding
MICU: Mobile intensive care unit
PPM: Personal protective materials
